# The effect of underlying inflammation on iron metabolism, cardiovascular risk and renal function in patients with type 2 diabetes

**DOI:** 10.1002/jha2.257

**Published:** 2021-07-06

**Authors:** Fransina Ndevahoma, Bongani B. Nkambule, Phiwayinkosi V. Dludla, Munyaradzi Mukesi, Kandiwapa N. Natanael, Tawanda M. Nyambuya

**Affiliations:** ^1^ Department of Health Sciences Faculty of Health and Applied Sciences Namibia University of Science and Technology Windhoek Namibia; ^2^ School of Laboratory Medicine and Medical Sciences College of Health Sciences University of KwaZulu‐Natal Durban South Africa; ^3^ Biomedical Research and Innovation Platform South African Medical Research Council Cape Town South Africa; ^4^ Department of Life and Environmental Sciences Polytechnic University of Marche Ancona Italy; ^5^ Division of Primary Healthcare at Katutura Community Health Centre Ministry of Health and Social Services Windhoek Namibia

**Keywords:** anaemia, cardiovascular risk, diabetic nephropathy, inflammation, type 2 diabetes

## Abstract

**Aim:**

To investigate the impact of inflammation on iron metabolism, cardiovascular risk and renal function in type 2 diabetes (T2D).

**Methods:**

A total of 50 patients with T2D were included in this study. The patients were stratified into two groups based on their levels of C‐reactive protein (CRP), namely normal and high levels (*n* = 25/group). All laboratory tests were measured using standardised methods.

**Results:**

Fasting plasma glucose levels were elevated in patients with high CRP when compared to those with normal levels (*p* = 0.0413). Total serum iron levels were lower in patients with high CRP levels (12.78 ± 3.50) when compared to those with normal levels (15.26 ± 4.64), *p* = 0.0381. However, ferritin and transferrin levels were comparable between the groups (*p* > 0.05). The mean cell volume (MCV) in the high CRP group was lower (87.66 ± 3.62) than the normal level group (90.79 ± 4.52), *p* = 0.0096, whilst the lipograms were similar (*p* > 0.05). The estimated glomerular filtration rate (eGFR) was lower in the high CRP group (98.06 ± 11.64) than the normal level group (104.7 ± 11.11), *p* = 0.046. Notably, CRP levels were negatively associated with serum iron levels (*r* = –0.38, *p* = 0.0061), MCV (*r* = –0.41, *p* = 0.0031), potassium (*r* = –0.37, *p* = 0.0086) and sodium levels (*r* = –0.28, *p* = 0.0471). Regression analyses showed that only CRP (*β* = –0.16, standard error [SE]: 0.06, *p* = 0.0125) and sodium (*β* = 0.51, SE: 0.25, *p* = 0.0434) levels contributed significantly to the prediction of serum iron levels.

**Conclusion:**

Underlying inflammation in T2D is associated with increased incidence of hypertension and reduced levels of serum iron, MCV and renal function. Although there was no apparent clinical anaemia or renal dysfunction in these patients, mitigating inflammation may be effective in circumventing the ultimate development of iron deficiency anaemia and chronic kidney disease in T2D.

## 1 INTRODUCTION

Type 2 diabetes (T2D) is amongst the leading non‐communicable diseases that are currently causing the most significant burden on the healthcare sector worldwide [1]. The recent International Diabetes Federation report estimated the global prevalence of T2D to be at 9.3% [2], with approximately two‐thirds of the cases being from low‐ to middle‐income countries. This high incidence of diabetes is ascribed to increased sedentary lifestyles and the eating of unhealthy diets, which both promote obesity and insulin resistance [3]. Notably, poor dietary choices are associated with iron deficiency, dyslipidaemia and cardiovascular disease (CVD) [4, 5].

T2D is a chronic inflammatory disorder that is linked with altered iron metabolism, with the iron stores strongly associated with glucose control [6, 7]. However, the exact pathological mechanisms that could explain altered iron metabolism in T2D remain elusive and are poorly understood. Nonetheless, obesity, a major risk factor for T2D and the use of glucose‐lowering drugs such as metformin have been implicated in dysmetabolic iron syndromes [8, 9]. In fact, anaemia caused by both iron deficiency and iron overload has been reported in patients living with T2D [10, 11]. A reciprocal relationship between low‐grade inflammation, obesity and iron disorders exists [12]. Low‐grade inflammation modulates the synthesis and action of hepcidin and erythropoietin, which both regulate iron metabolism denoted by the serum iron profiles [13]. For instance, aberrant levels of total serum iron, iron stores and transferrin, an iron transporter protein modulated by these regulators have been described in patients with T2D [14, 15, 16]. This alteration in iron metabolism usually leads to anaemia, a symptom that is closely associated with dyslipidaemia [17, 18], one of the major hallmarks of T2D and CVD [19, 20]. Although the exact inferences on the relationship between iron metabolism and poor glucose control are controversial and influenced by various factors, it is evident that they are closely connected and need further exploration.

Type 2 diabetes is an independent risk factor for chronic kidney disease [21]. In fact, over 40% of patients with T2D develop diabetic nephropathy (DN), a condition that is characterised by proteinuria or reduced renal function in their lifetime [22]. The high incidence of DN in T2D has resulted in excessive mortality in these patients despite intensive therapies that alleviate its risk factors such as hyperglycaemia, hypercholesterolemia and hypertension [23]. Notably, a recent body of evidence has suggested the involvement of inflammation in the pathogenesis of DN and has sparked interest in the exploration of anti‐inflammatory drugs as a therapeutic strategy in preventing the manifestation of DN in these patients [24, 25, 26]. We, therefore, questioned whether the degree of inflammation can be used to stratify DN risk in patients with T2D. Thus, this study first aimed to investigate the effect of inflammation on iron profiles and their associated haematological indices in patients with T2D. Second, we intended to assess the cardiovascular risk and renal function in T2D as well as determining any associations between inflammatory indices, renal function tests, iron and lipid profiles in patients with T2D.

## 2 MATERIALS AND METHODS

### 2.1 Study population

This cross‐sectional study involved a cohort of outpatients with clinically known cases of T2D from Khomas urban areas that visited the Katutura Community Health Centre, Windhoek, Namibia between September and December 2020. Informed consent was sought from all participants and the research was conducted in accordance with the Declaration of Helsinki (2008) of the World Medical Association. The study was approved by the Namibia University of Science and Technology Research Ethics committee (FHAS 1/2020) and the Namibia Ministry of Health and Social Services (17/3/3 MN). A total of 115 adult patients self‐reported as Black were enrolled, and only 50 were included in this study after the minimum sample size calculation. The included cases of T2D were clinically diagnosed using the American Diabetes Association guidelines [27]. All patients underwent a general physical examination prior to enrolling in the study. We excluded patients with active or recent infections, pregnant patients and those under the age of 18 years. We used serum c‐reactive protein (CRP) levels to stratify the included patients into two groups based on their inflammatory state. The normal CRP group consisted of patients with CRP levels of 1–≤10 mg/L, and a high CRP group with CRP levels of >10 mg/L, which was indicative of an underlying inflammation.

### 2.2 Laboratory measurements

Blood samples for analysis were collected by a trained nurse into EDTA and SST vacutainers tubes for analysis at an ISO 15189 of 2012 accredited laboratory (Namibia Institute of Pathology, Windhoek, Namibia). In order to determine glycaemic control in the included patients, we measured the levels of fasting plasma glucose (FPG) and glycated haemoglobin using the Cobas c501 analyser (Roche, Basel, Switzerland) as per the manufacturer's instructions. The levels of CRP and the erythrocyte sedimentation rate (ESR) were evaluated using the Alinity c analyser (Abbot, Illinois, USA) and Test 1 THL Alifax S.p.A (Alifax, Udine, Italy), respectively, to determine patients’ inflammatory state and their subsequent stratification. To investigate the effect of underlying inflammation on iron metabolism in T2D, we measured the iron profiles (serum iron, transferrin and ferritin) using the Alinity c analyser (Abbot). In addition, we evaluated the red cell indices that are dependent on iron metabolism by performing complete blood cell counts using a Sysmex 1000 XN automated haematology analyser (Sysmex Corporation, Kobe, Japan). As part of the blood cell count, we also assessed white cell and platelet count and determined the levels of total protein, globulins and albumin, a negative acute‐phase protein [28] as surrogate markers of inflammation using the Alinity c analyser (Abbot). To assess the impact of inflammation on renal function, we measured the levels of potassium, sodium, urea and creatine using the Anility c analyser (Abbot) and further calculated the estimated glomerular filtration rate (eGFR) using the modification of diet in renal disease study's equation (MDRD) [29]. Finally, to stratify the cardiovascular risk in the included patients, we performed lipid measurements (cholesterol levels and triglycerides) using the Alinity c analyser (Abbot).

### 2.3 Statistical methods and data analysis

The sample size was calculated using G*Power software (Version 3.1.9.2) based on the effect size of the primary ouctome reported in a previous study [30]. The following assumptions were used in determining the minimum number of required participants: a medium effect size (*d*) = 0.898071, *α*_err_ prob =  0.05, power (1‐*β*_err_ prob) = 0.80 and an allocation ratio of 1:1. The D'Agostino & Pearson test was performed for normality testing. A chi‐square test was used to test for relationships between categorical variables. Data were reported as mean ± SD or median and interquartile range depending on the data distribution. For parametric data, the unpaired two‐tailed student's *t*‐test was used and in cases of unequal variance, a Welch's correction was performed. The Mann‐Whitney *U* test was used to compare non‐parametric data. Bivariate correlations were performed using the Spearman coefficient and the multivariant regression analysis was conducted to explain the relationship between total serum iron, CRP and FPG, eGFR, potassium and sodium levels. A *p*‐value < 0.05 represented statistical significance. All statistical analysis was performed using GraphPad Prism 8 version 8.0.2 Software (GraphPad Software Inc, San Diego, CA, USA).

## 3 RESULTS

A total of 50 adult patients with T2D were included in this study, 25 with normal and 25 with high CRP levels. The demographic and clinical characteristics of the included participants are shown in Table 1. The groups had a similar age distribution and the patients were from a similar socio‐economic and ethnic background as they were recruited from the same community. All included patients were Blacks (Africans) and had a mean age of 50.16 ± 12.72 years and a male to female ratio of 0.43.

**TABLE 1:** Clinical characteristics and laboratory profiles of included patients (*n* = 50)

Parameter	T2D with normal CRP (*n* = 25)	T2D with high CRP (*n* = 25)	*p*‐value
Clinical characteristics			
Age (years)	50.64 ± 13.63	49.68 ± 12.01	0.7927
Male, *n* (%)	7 (28)	8 (32)	0.7576
Body mass index (kg/m^2^)	28.13 ± 4.94	30.43 ± 5.62	0.1394
Systolic blood pressure (mm/Hg)	137.5 ± 21.83	137.4 ± 20.18	0.9833
Diastolic blood pressure (mm/Hg)	84.13 ± 11.38	86.39 ± 13.00	0.5334
Hypertension, *n* (%)	13 (52)	16 (64)	0.3900
Duration of T2D (years)	5.00 (2.00–12.00)	4.00 (1.00–13.00)	0.9807
Metformin, *n* (%)	18 (72)	17 (68)	0.7576
Insulin, *n* (%)	0	4 (16)	**0.0371**
Metformin and insulin, *n* (%)	7 (28)	4 (16)	0.3050
Glucose profiles			
Glycated haemoglobin (%)	8.500 (6.20 – 9.90)	8.700 (7.20 – 9.40)	0.6125
Fasting plasma glucose (mmol/L)	8.86 ± 3.68	11.12 ± 3.95	**0.0413**
Inflammatory profiles			
C‐reactive protein (mg/L)	3.87 ± 2.56	17.06 ± 7.88	**<0.0001**
ESR (mm/h)	22.60 ± 19.45	44.57 ± 32.13	**0.0114**
White cell count (10^9^/L)	6.77 ± 1.49	7.11 ± 1.99	0.4922
Platelet count (10^9^/L)	309.2 ± 81.06	327.9 ± 87.21	0.4357
Albumin (g/L)	42.93 ± 3.31	41.84 ± 2.98	0.2282
Total protein (g/L)	78.80 ± 5.07	82.38 ± 5.06	**0.0160**
Globulin (g/L)	36.45 ± 3.86	40.65 ± 3.79	**0.0003**
Albumin/globulin ratio	1.19 ± 0.18	1.04 ± 0.13	**0.0013**
Lipid profiles			
Triglycerides (mmol/L)	1.67 ± 0.68	1.62 ± 0.69	0.8042
Total cholesterol (mmol/L)	4.91 ± 1.13	4.59 ± 0.77	0.2481
LDL–cholesterol (mmol/L)	3.10 ± 1.04	2.80 ± 0.87	0.2617
HDL–cholesterol (mmol/L)	1.02 ± 0.27	1.06 ± 0.32	0.6419
HDL–cholesterol ratio	0.22 ± 0.06	0.23 ± 0.07	0.5313

Abbreviations: CRP, C‐reactive protein; ESR, erythrocyte sedimentation rate; HDL, high‐density lipoprotein; LDL, low‐density lipoprotein; T2D, type 2 diabetes.

Results expressed as mean ± standard deviation and median interquartile range.

### 3.1 Clinical parameters and glucose parameters

There were no significant differences in the body mass index, systolic blood pressure, diastolic blood pressure and disease duration between the two groups (*p* > 0.05) (Table 1). Hypertension was more prevalent in patients with underlying inflammation (OR = 1.64, 95% CI: [0.53, 5.09]). Whereas the levels of glycated haemoglobin were comparable between the two groups (*p* > 0.05), while FPG levels were higher in T2D with underlying inflammation (11.12 ± 3.95) when compared to patients with T2D with normal CRP levels (8.86 ± 3.68), *p* = 0.0413 (Table 1). A total of 70% of the included patients were on metformin treatment, whilst 8% were on insulin and 22% were on a combination of metformin and insulin (Table 1).

### 3.2 Inflammatory profiles

The levels of CRP were used as a dependent factor to stratify the patients based on their inflammatory status. Similarly, the ESR (*p* = 0.0114), total protein (*p* = 0.0160) and globulin levels (*p* = 0.0003) were elevated in the patients with T2D and high CRP levels when compared to those with normal CRP levels (Table 1). However, there were no significant differences in the white cell, and platelet counts and albumin, the negative acute‐phase reactant between the two groups (*p* > 0.05) (Table 1).

### 3.3 Lipid profile levels

Dyslipidaemia is closely associated with increased cardiovascular risk in patients with T2D [31]. Therefore, we measured lipograms in patients with T2D. The levels of triglycerides, total cholesterol (Tc), low‐density lipoprotein (LDL)‐c, high‐density lipoprotein (HDL)‐c and HDL/cholesterol ratio were comparable between the two groups (*p* > 0.05) (Table 1).

### 3.4 Iron profile levels and red blood cell indices

**FIGURE 1:**
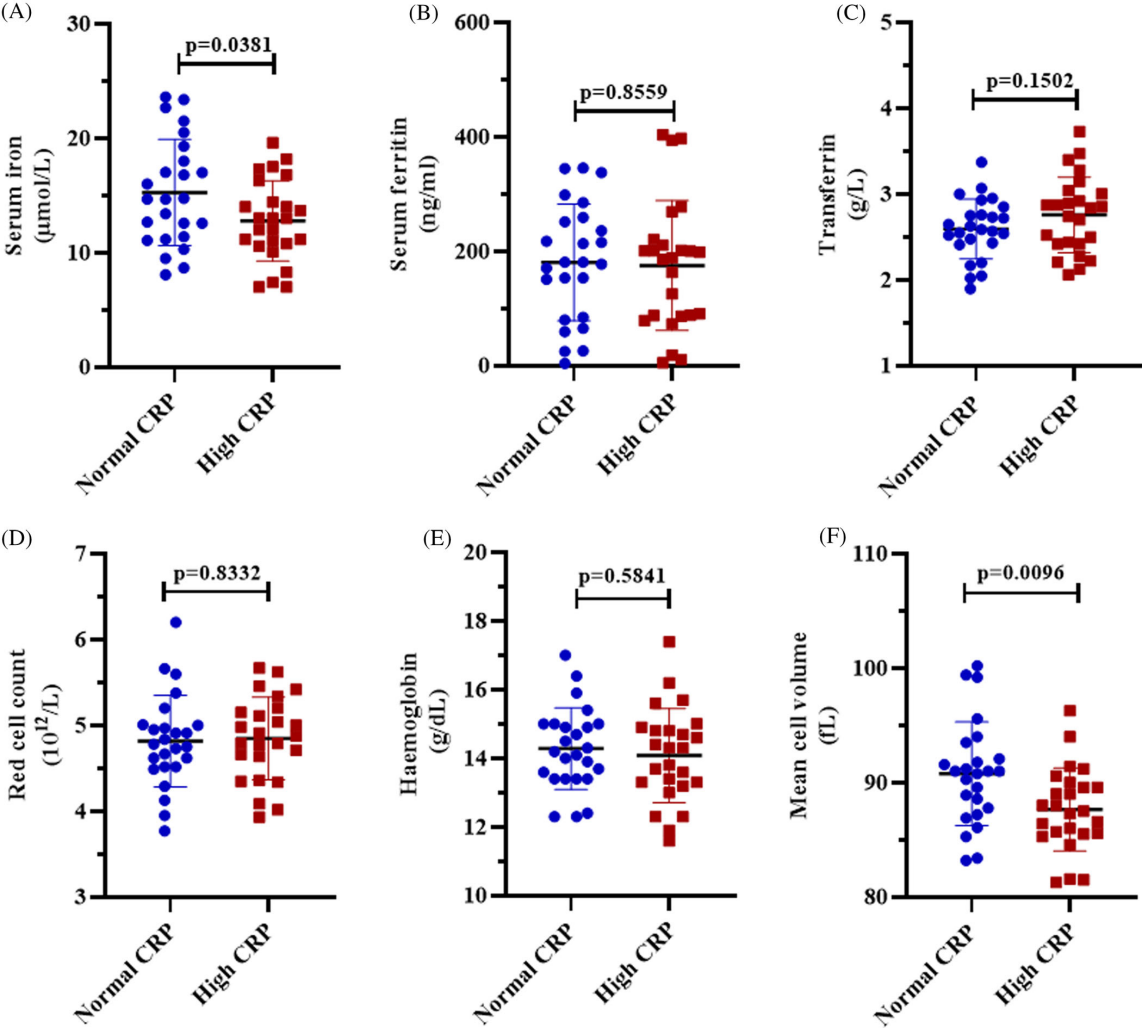
A comparison of iron profiles between patients with normal and high c‐reactive protein levels. The levels of serum iron (A) and red cell mean volume (F) were significantly lower in patients with underlying inflammation when compared to those without. However, comparable levels of ferritin (B) and transferrin (C) as well as red cell count (D) and haemoglobin (E) were observed between the two groups. All results were expressed as mean ± standard deviation

In order to assess the impact of inflammation on iron metabolism, we measured iron profiles in patients with T2D. Patients with T2D and high CRP levels had lower levels of total serum iron (12.78 ± 3.50) in comparison to patients with normal CRP levels (15.26 ± 4.64), (*p* = 0.0381; Figure 1A). However, there were no differences in the levels of ferritin and transferrin between the two groups (*p* > 0.05; Figure 1B–C, Table 2).

**TABLE 2:** Iron profiles and its associated indices in patients with type 2 diabetes (T2D; *n* = 50)

Parameter	T2D with normal CRP (*n* = 25)	T2D with high CRP (*n* = 25)	*p*‐value
Iron profiles			
Serum iron (μmol/L) Ferritin (ng/mL)	15.26 ± 4.64 180.9 ± 102.1	12.78 ± 3.50 175.3 ± 113.4	**0.0381** 0.8559
Transferrin (g/L)	2.59 ± 0.35	2.76 ± 0.44	0.1502
Red cell indices			
Red cell count (10^12^/L)	4.82 ± 0.53	4.85 ± 0.48	0.8332
Haemoglobin (g/dl)	14.28 ± 1.19	14.08 ± 1.37	0.5841
Haematocrit (%)	42.74 ± 5.17	42.48 ± 4.19	0.8482
Mean cell volume (fL)	90.79 ± 4.52	87.66 ± 3.62	**0.0096**
MCH (pg)	29.32 ± 1.49	28.64 ± 1.44	0.1057
MCHC (g/dL)	32.90 ± 1.08	32.94 ± 0.91	0.8878
Red cells distribution width (%)	13.33 ± 1.12	13.40 ± 0.86	0.8108
Kidney function tests			
Potassium (mmol/L)	4.64 ± 0.37	4.36 ± 0.28	**0.0042**
Sodium (mmol/L)	137.4 ± 2.57	135.4 ± 1.49	**0.0017**
Urea (mmol/L)	4.03 ± 1.10	4.51 ± 1.42	0.1844
Creatinine (μmol/L)	81.16 ± 17.32	74.53 ± 10.17	0.1066
eGFR MDRD (ml/min/1.73 m²)	104.7 ± 11.11	98.06 ± 11.64	**0.0461**

**Abbreviations**: CRP, C‐reactive protein; eGFR MDRD, estimated Glomerular Filtration Rate based on Modification of Diet in Renal Disease Study's equation; MCH, mean corpuscular haemoglobin; MCHC, mean corpuscular haemoglobin concentration; T2D, type 2 diabetes.

We further measured haematological indices that are closely associated with iron metabolism. Although the red blood cell count and haemoglobin levels were comparable between the two groups (*p* > 0.05) (Figure 1D–E), the red cell mean volume (MCV) in patients with T2D and high CRP levels was lower (87.66 ± 3.62) than in patients with normal CRP levels (90.79 ± 4.52), (*p* = 0.0096; Figure 1F). All other red blood cell indices were comparable between the two groups (*p* > 0.05; Table 2).

### 3.5 Renal function tests

DN is one of the most prevalent T2D‐associated complications, that promotes the pathogenesis of chronic kidney disease [32, 33]. Therefore, we assessed kidney function tests in the included patients and further assessed whether the renal impairment is exacerbated by underlining inflammation. Notably, patients with T2D and high CRP levels showed reduced levels of potassium (*p* = 0.0042), sodium (*p* = 0.0017) and eGFR MDRD (*p* = 0.0461) when compared to those with normal CRP levels (Table 3). Despite these differences, the levels were within the normal range. The levels of creatinine (*p *= 0.1066) and urea (*p* = 0.1844) were comparable between the two groups (*p* > 0.05; Table 2).

**TABLE 3:** Multivariate linear regression analysis of predictors of serum total iron, estimated glomerular filtration rate and c‐reactive protein levels in type 2 diabetes

Total iron levels			
Variable	Beta	Standard error	*p*‐value
C‐reactive protein	–0.16	0.06	**0.0125**
Fasting plasma glucose	0.23	0.14	0.1052
Sodium	0.51	0.25	**0.0434**

Estimated glomerular filtration rate			
Variable	Beta	Standard error	*p*‐value
C‐reactive protein	–0.34	0.20	0.1003
Total iron levels	–0.69	0.44	0.1265
Fasting plasma glucose	–0.34	0.43	0.4367
Sodium	1.02	0.77	0.1944

C‐reactive protein			
Variable	Beta	Standard error	*p*‐value
Total iron levels	–0.74	0.28	**0.0115**
Fasting plasma glucose	0.23	0.30	0.4432
Potassium	‐5.64	3.42	0.1066

### 3.6 Correlation and regression analysis of glucose levels, CRP and iron profiles

To determine whether there are any associations between glucose, inflammation, iron profiles and renal function in patients with T2D, we performed a multivariate correlation analysis. Notably, the CRP levels were negatively associated with total serum iron levels (*r* = –0.38, *p* = 0.0061), MCV (*r* = –0.41, *p* = 0.0031), potassium (*r* = –0.37, *p* = 0.0086) and sodium (*r* = –0.28, *p* = 0.0471). As expected, the levels of CRP also correlated with other inflammatory profiles, namely ESR (*r* = 0.54, *p* < 0.0001), globulin (*r* = 0.45, *p* = 0.0010) and A/G ratio (*r* = –0.47, *p *= 0.0006). However, there were no correlations between FPG and iron profiles or renal function (*p* > 0.05). We further assessed the significant predictors of iron levels, CRP, FPG and eGFR in patients with T2D using multivariate regression analyses. Notably, CRP (*β *= –0.16, SE: 0.06, *p* = 0.0125) and sodium levels (*β* = 0.51, SE: 0.25, *p* = 0.0434) significantly influenced total iron levels whilst eGFR levels could not be predicted by CRP, total iron levels, FPG or sodium levels (*p* > 0.05; Table 3).

## 4 DISCUSSION

This study aimed at investigating the impact of inflammation on iron metabolism, renal function, and cardiovascular risk in patients with T2D. Our results showed reduced total serum iron levels and red cell mean volume in patients with underlying levels of inflammation. Notably, treatment with metformin, insulin, or a combination of both was associated with failure to normalise glucose levels in all patients and the hyperglycaemia observed was more pronounced in patients with T2D and high CRP levels. Moreover, all included patients had high blood pressure and the prevalence of overt cases of hypertension were marked in T2D with high CRP levels. Thus, these findings highlight the impact of inflammation in impairing glucose control and increasing cardiovascular risk. In the context of the latter, our findings did not show any cardio‐protective effects of metformin, a first‐line oral anti‐hyperglycaemic drug. Although a negative association between iron levels and lipid profiles (triglycerides, Tc and LDL‐c) has been previously described [34], the current study found no associations between these parameters. Finally, the levels of sodium, potassium and eGFR were lower in T2D with high CRP levels, and this is indicative of impaired renal function. Notably, this dysfunction was only dependant on the degree of inflammation and independent of glucose control or iron metabolism.

Low‐grade inflammation in T2D modulates the synthesis of hepcidin, one of the important regulators of iron metabolism. Hepcidin maintains iron homeostasis through the inhibition of iron absorption and release from the intestines and macrophages as well as its subsequent transportation to the bone marrow for erythropoiesis [35]. The synthesis of iron is dependent on the inflammatory status and the oxygen‐carrying capacity in the body [12, 36]. In that context, the increased release of interleukin (IL)‐6 during inflammation induces the activation of the Janus kinase (JAK)/signal transducer and activator of transcription 3(STAT3) proteins signalling which activates the transcription of the *HAMP* gene [37]. Whereas, an increase in body iron stores activate the bone morphogenetic protein/s‐mothers against the decapentaplegic transduction pathway which induces the downstream activation of *HAMP* gene and the translation of hepcidin thereof [38]. The synthesis and action of hepcidin is also modulated by erythropoietin, a hormone that is released by the kidney in anaemic hypoxia to initiate red cell synthesis [13, 39]. The dysregulation of these important hormones alters iron metabolism leading to the manifestation of anaemia in T2D [13]. Although the patients included in this cohort were not anaemic, the reduced levels of total serum iron levels and MCV suggest the early onset of microcytic iron deficiency anaemia in patients with T2D coupled with underlying inflammation. Therefore, alleviating inflammation in T2D could aid in improving iron metabolism in patients with a significant underlying inflammation since CRP and ESR levels were negatively associated with decreased total serum iron and MCV. Apart from the aberrant expression of iron regulator proteins, the reduction in total serum iron levels may be due to the side effects of metformin treatment since it is widely accepted to cause vitamin B12 malabsorption and its subsequent deficiency in circulation, resulting in impaired erythropoiesis [40].

It is widely acknowledged that obesity alters glucose metabolism and predisposes patients with T2D to develop CVD [41]. In fact, about a third of patients with T2D have CVD [42]. Notably in our study, patients with T2D and high CRP levels were associated with class I obesity and poor glucose control as denoted by a body mass index  > 30 kg/m^2^and elevated FPG levels, respectively. These findings further highlight the effect of inflammation in aggravating poor glucose control in T2D via the activation of various pathways and the impairment of insulin signalling as previously reviewed [43]. This association has been ascribed to exacerbated inflammation and altered lipid metabolism which causes atherosclerosis and hypertension [44]. Although the blood pressure was comparable between the two groups, it is evident that hypertension was more prevalent in patients with T2D and high CRP levels. Thus, further highlighting the impact of obesity‐associated inflammation on cardiovascular risk. Therefore, the use of anti‐inflammatory drugs such as low‐dose aspirin in patients with T2D is important in reducing cardiovascular risk in these patients as previously described [45].

In addition to other factors associated with poor glucose control and obesity, dyslipidaemia promotes the initiation of atherosclerosis and arterial thrombosis, which are both major risk factors for the pathogenesis of CVD in patients with T2D [46]. Although triglycerides and cholesterol levels were comparable between the groups, elevated triglycerides, Tc, and LDL‐c coupled with reduced HDL‐c levels in patients with T2D are associated with increased cardiovascular risk [47]. Therefore, cholesterol‐lowering drugs such as statins are recommended for the primary prevention of CVD in patients with T2D [48].

DN is closely associated with systemic inflammation mediated by exacerbated activation of the JAK/STAT signalling pathway, the transcription factor nuclear factor‐kB, and inflammatory cytokines [24, 49, 50]. The resulting pro‐inflammatory milieu promotes the migration and infiltration of immune cells, particularly macrophages, into renal tissue [49]. Once activated, macrophages release pro‐inflammatory cytokines such as IL‐1, tumour necrosis factor‐ α and IL‐6 which overall induces renal hypertrophy [25]. In addition, IL‐6 alters the permeability of the glomerular endothelium and thickens the glomerular basement membrane, resulting in reduced GFR [25, 50]. Interestingly, IL‐6 stimulates the release of CRP, hence both are used as well‐established inflammatory biomarkers. Notably, it was evident that patients with high levels of CRP had lower eGFR and imbalanced electrolyte levels, thus highlighting the negative impact of inflammation on renal function. Although the renal function influences iron regulation by secreting erythropoietin, we did not find any association between its function and iron profiles. In all, the current study had limitations, as we did not assess the levels of regulators of iron metabolism such as hepcidin and erythropoietin. However, elevated levels of hepcidin and reduced erythropoietin levels, the hallmark of inflammation‐induced iron deficiency anaemia, were reported in patients with T2D [11, 51, 52].

## 5 CONCLUSION

Iron metabolism is significantly influenced by the inflammatory status in patients with T2D. As such, reduced total serum iron levels and MCV are features of underlying inflammation in T2D. Moreover, the presence of underlying inflammation in these patients is closely associated with an increased incidence of hypertension and reduced renal function. Therefore, the amelioration of inflammation in patients with T2D may be an effective intervention to lower cardiovascular risk and circumvent the ultimate development of iron deficiency anaemia and chronic kidney disease in these patients.

## CONFLICT OF INTEREST

The authors declare no conflict of interest.

## AUTHOR CONTRIBUTIONS

Fransina Ndevahoma, Bongani B. Nkambule and Tawanda M. Nyambuya conceptualized, designed the study and drafted the manuscript. Fransina Ndevahoma, Bongani B. Nkambule and Tawanda M. Nyambuya performed formal analysis, methodology and validation as well as visualization. All authors including Phiwayinkosi V. Dludla, Munyaradzi Mukesi and Kandiwapa N. Natanael wrote, reviewed, edited and approved the final manuscript.

## FUNDING INFORMATION

Namibia University of Science and Technology (NUST), Grant Number: MG/2017; NUST Postgraduate Research Fund.
